# Predicting the Trajectories of Parents’ Relationship Well-Being During COVID-19 Lockdowns and Beyond: a Vulnerability, Stress and Adaptation Model Perspective

**DOI:** 10.1007/s11121-023-01498-1

**Published:** 2023-03-28

**Authors:** Gery C. Karantzas, John W. Toumbourou, Laura Knox, Christopher C. Greenwood, Elizabeth M. Westrupp

**Affiliations:** 1 School of Psychology, Deakin University, Geelong, VIC 3220 Australia; 2 Centre for Social and Early Emotional Development, Deakin University, VIC Geelong, 3220 Australia

**Keywords:** COVID-19, Lockdown, Parents, Relationship satisfaction, Loneliness

## Abstract

Strict lockdowns have been employed by many of the world’s nations as a public health response to COVID-19. However, concerns have been expressed as to how such public health responses disturb the human ecosystem. In this paper, we report on findings from a longitudinal study of Australian parents in which we investigated how state differences in government-mandated lockdowns affect the relationship well-being (i.e., relationship satisfaction and loneliness) of parents. We situated the study of the relational effects of strict lockdowns within the Vulnerability Stress Adaptation Model (VSAM, Karney & Bradbury, 1995) that considers the role of parents’ pre-existing vulnerabilities (i.e., psychological distress and attachment insecurity), life stressors (pre-pandemic and COVID-19 stressors), and adaptive relationship processes (constructive communication and perceived partner support). A total of 1942 parents completed 14 waves of assessments of relationship satisfaction and loneliness over a 13.5-month period as well as baseline assessments of personal vulnerabilities, life stressors, and adaptive relationship processes. Parents with high relationship adaptations and low vulnerabilities evidenced the highest relationship well-being (i.e., high satisfaction and low loneliness) during changes in lockdown restrictions, while parents with moderate relationship adaptations and vulnerabilities experienced the poorest well-being. Differences in state lockdown restrictions (i.e., Victoria [long and strict lockdown policy] vs all other states) were associated with differences in relationship well-being for parents with high relationship adaptations. Specifically, Victorian parents experienced significant declines in relationship well-being compared to non-Victorian parents. Our findings provide novel insights into how government-mandated social restrictions can disrupt the relational ecology of parents.

**Supplementary Information:**

The online version contains supplementary material available at 10.1007/s11121-023-01498-1.

Over the course of the pandemic, concerns have been raised regarding the relationship well-being (i.e., relationship satisfaction and loneliness) of world’s citizens. This concern has emerged due to the assumed social disconnection and isolation brought on by government-mandated lockdowns (policies requiring sustained residential containment, also referred to as “stay-at-home orders”). Specifically, strict lockdowns are argued to disturb the human ecosystem (Borkowska & Laurence, 2021)—an ecosystem underpinned by an evolutionary basis for human bonding and social affiliation (Bowlby, 1969/1982; Caporael & Brewer, 1991).

Research into the relationship well-being of people during COVID-19 is increasingly focusing on the romantic relationships of parents (e.g., Russell et al., 2021); however, studies have placed little emphasis on investigating the effects of lockdown. Understanding the relational toll of social restrictions on parents is particularly important given that the increased responsibilities that have fallen on parents have likely compromised the time and effort that parents can devote towards their relationships (McCrae et al., 2021; Westrupp et al., 2022a, b).

The Australian experience of the COVID-19 pandemic represents a unique natural experiment to investigate how differences in the length and strictness of social confinement can affect parent’s trajectory of relationship well-being. This is because although all of Australia experienced to a 2-month lockdown, the state of Victoria, Australia’s second most populated state, endured a second, and stricter lockdown as Victoria underwent a second wave of COVID-19. During this period, the rest of Australia experienced the easing of restrictions. Thus, exploring the differences amongst parents who resided in Victoria, compared to other parts of Australia, can provide important insights into how differences in social confinement affect parental relationship well-being across the pandemic.

However, research into relationships over the last three decades suggests that not all parents experience the same trajectory in terms of their relationship well-being during times of crises and disasters (e.g., Fredman et al., 2010; Karantzas et al., 2022). Thus, differences in the length and strictness of lockdowns are unlikely to have a uniform effect on the romantic relationship well-being of parents over time. To date, research into the science of relationships has highlighted that a raft of individual, contextual, and interpersonal factors can affect the trajectory of relationship well-being in romantic relationships (Karney & Bradbury, 1995; Righetti et al., 2022). But who are the parents who are most at risk of experiencing declines in their romantic relationships during periods of social confinement? An important framework for understanding how individual, contextual, and interpersonal factors contribute to stability and change in relationship well-being during strict lockdowns is the Vulnerability Stress Adaptation Model (VSAM, Karney & Bradbury, 1995).

The VSAM outlines how stressors, along with key enduring personal vulnerabilities and relationship adaptive factors, converge to explain the trajectory of relationship wellbeing. In the current paper, we draw on the VSAM to investigate whether parents with different profiles of personal vulnerabilities, stressors, and relationship adaptations near the beginning of the pandemic, experience different trajectories of relationship well-being, and whether these trajectories differed for Victorian and non-Victorian parents.

Our investigation draws on data from the COVID-19 Pandemic Adjustment Survey (CPAS, Westrupp et al., 2020), a longitudinal study that tracked the personal and relational well-being of parents of a child 0–18 years over a 14-month period. In the current paper, we focus on the subsample of parents who were currently in a romantic relationship. These parents completed multiple assessment waves of relationship well-being during which time Victorian and non-Victorian parents experienced differences in social confinement.

Given that current study is situated within the context of COVID-19 lockdown orders that differed between Australian parents residing in and out of Victoria, we begin by providing a brief description regarding the nature and timeline of the activation and deactivation of government-mandated lockdowns in Australia across 2020. We then provide a brief description of the VSAM and the key variables that are assumed to play an important role in predicting relationship wellbeing during the COVID-19 crisis before outlining our study aims.

## COVID-19 Social Restrictions: the Australian Experience

During the first year of the pandemic, Australia, like many parts of the world, experienced a nation-wide lockdown to curb the spread of COVID-19. By April 2020, all Australian states and territories had declared a state of emergency and a government-mandated level 3 national lockdown (i.e., “stay-at-home” orders) was enforced. During the national lockdown, people living in Australia were mandated to home confinement, except for four reasons: (1) shopping for food and supplies, (2) receiving care and caregiving, (3) time-limited exercise, and (4) study or work—if unable to do so from home (Department of Health, 2020). Australians experienced an 8-week lockdown from April through to May. From June of 2020, most Australian states exited lockdown and enforced minimal social restrictions due to the return to low infection rates in the community. However, Victoria, Australia’s second most populous state (6.4 million citizen’s, 25% of Australia’s population) experienced a second wave of the COVID-19 pandemic activating a second and stricter lockdown (i.e., level 4) that extended to the closure of early childhood education and care services. At the time, the Victorian lockdown would become one of the longest and strictest in the world, spanning four months from June to October 2020. In total, Victorians experienced “stay at home” orders for 176 days compared to the 56 days experienced by non-Victorians. This striking contrast of days in lockdown between Victorian and non-Victorians provides a rare opportunity to investigate the effects of social confinement on the trajectory of relationship well-being in parents who differ in their profile of personal enduring vulnerabilities, external life stressors, and adaptive relationship processes.

## The Vulnerability Stress Adaptation Model (VSAM)

The VSAM is a broad theoretical model of relationship stability and change (Karney & Bradbury, 1995) that has recently been applied and extended to understand the trajectory of romantic relationships during COVID-19 pandemic (Karantzas et al., 2022; Pietromonaco & Overall, 2021, 2022). According to the VSAM, the trajectory of relationship well-being is related to the interplay of three sets of factors – *enduring personal vulnerabilities, external stressors, and adaptive relationship processes* (Karantzas et al., 2022; Karney & Bradbury, 1995; Pietromonaco & Overall, 2021, 2022).

*Enduring personal vulnerabilities* reflect characteristics and aspects of a person’s disposition that challenges an individual’s capacity to function effectively within their romantic relationship (Karantzas et al., 2022; Karney & Bradbury, 1995; Pietromonaco & Overall, 2021, 2022). *External life stressors* reflect pressures that compromise a couple’s ability to maintain a loving and satisfying relationship. These stressors can be physical, social, cultural, and historical in nature. Such stressors include (but are not limited to) financial strain, job instability or loss, threats to physical health, pressure to maintain particular social roles and responsibilities, racism/discrimination, and difficulties with family and friends. For parents, a central stressor is the role of parenthood itself, in which parents must balance the multiple responsibilities of child rearing and deal with sleep deprivation during the early years of parenthood (Westrupp et al., 2022a, b; in press). *Adaptive relationship processes* reflect the cognitions and behaviors that foster relationship positivity or reduce relationship negativity (Karantzas et al., 2022; Karney & Bradbury, 1995; Pietromonaco & Overall, 2021).

### Enduring Personal Vulnerabilities

The most commonly theorized and researched enduring personal vulnerability factors are pre-existing symptoms of psychological distress (namely, symptoms of depression, anxiety, and stress) and attachment insecurity (i.e., chronic anxiety and/or discomfort within close relationships) (Karantzas et al., 2022). In terms of psychological distress, various theoretical and clinically focused models of relationship functioning make a case for the negative effects that enduring mental health problems and psychological distress can have on relationship outcomes (e.g., Beach & Cassidy, 1991; Braithwaite & Holt-Lunstad, 2017; Karney & Bradbury, 1995). Moreover, such vulnerabilities have been found to increase in severity during the COVID-19 pandemic (e.g., Pierce et al., 2021; Westrupp et al., 2021), and especially during periods of strict social restrictions (Fancourt et al., 2021; Knox et al., 2022; Westrupp et al., 2021). In particular, the sense of hopelessness and lack of efficacy associated with depression, and the chronic feelings of fear and worry that characterize anxiety, can contribute to the development and maintenance negative relationship biases and a diminished capacity to perspective-take and problem-solve relationship challenges and issues (Hammen, 1991).

Attachment insecurity (i.e., chronic worry and distrust of romantic partners) has also been found to be consistently associated with experiencing relationship dissatisfaction and loneliness (see Gillath et al., 2016; Mikulincer & Shaver, 2016 for reviews) that is exacerbated in times of distress and crises which can extend to the COVID-19 pandemic (Karantzas et al., 2022; Pietromonaco & Overall, 2021; Overall et al. 2022). Research into romantic relationships suggests that attachment insecurity is underpinned by two related but distinct dimensions (Brennan et al., 1998; Gillath et al., 2016; Mikulincer & Shaver, 2016). The first is attachment anxiety and is characterized by an excessive need for approval, preoccupation with relationship partners, and chronic fear of abandonment (Brennan et al., 1998; Fraley et al., 2000; Karantzas et al., 2010). The second is attachment avoidance and is characterized by a distrust of relationship partners, discomfort with relationship closeness, and excessive self-reliance (Brennan et al., 1998; Fraley et al., 2000; Karantzas et al., 2010). Those who experience attachment insecurity (much like those who experience psychological distress) tend to hold quite negative relationship cognitions and views of the self as well as romantic partners. Consequently, insecurely attached individuals are pessimistic about a partner’s ability to meet their personal and relationship needs and can respond to partners in destructive ways (e.g., hostility, withdrawal) that reduce relationship satisfaction and heighten loneliness (e.g., Feeney, 2016; Gillath et al., 2016; Karantzas et al., 2014).

### External Life Stressors

A number of external life stressors have been found to place strain on romantic relationships (e.g., Neff & Karney, 2017; Randall & Bodenmann, 2017). During the COVID-19 pandemic, many parents entered the early stages of the COVID-19 pandemic with pre-existing life stressors (e.g., Evans et al., 2020) related to financial and work stress of parenthood itself (Evans et al., 2020; Westrupp et al., 2022a, b). However, the social and economic disruption brought on by government-mandated social restrictions meant that many citizens of countries all over the world experienced the sudden onset of multiple significant stressors including job and financial insecurity, housing insecurity, and for many parents, the need to facilitate home schooling while also juggling work and other responsibilities. These stressors are considered to tax parent’s regulatory capacities to respond effectively and constructively in times of family difficulties which can heighten problems, dissatisfaction, and feelings of disconnection (Karantzas et al., 2022).

### Adaptive Relationship Processes

Two of the most widely studied relationship adaptations are constructive communication strategies and perceived partner support (Karantzas et al., 2022; Karney & Bradbury, 1995; Pietromonaco & Overall, 2021). Constructive communication strategies that entail positive verbal and non-verbal self-expression (e.g., communicating praise, expressing gratitude/admiration) and the inhibition of negative self-expression (e.g., anger, hostility) contribute to the validation and appreciation of one’s partner and to the clear discussion of relationship challenges, issues, and responsibilities while regulating one’s emotions (Overall & McNulty, 2017; Sillars & Vangelisti, 2006). It is therefore not surprising that constructive communication has been found to enhance relationship satisfaction and decrease loneliness (e.g., Yum, 2003). In the context of COVID-19, the use of constructive communication by parents is likely to be an important adaptive factor in the maintenance of relationship satisfaction and in reducing feelings of loneliness. This is because constructive communication facilitates clear communication of needs and concerns as to how parents can support one another’s socio-emotional well-being in dealing with COVID-19 stressors as well as the challenges of stay-at-home orders.

Similarly, perceiving support by a romantic partner during difficult and challenging life events and crises buffers the stress of adverse life events experienced by parents (Cutrona & Russell, 1987, 2017). Specifically, parents that recognize that the support provided by one’s partner is responsive and sensitive to one’s needs ensures that the support-recipient feels understood, validated (Cutrona & Russell, 1990; Reis & Clark, 2013), and satisfied within their romantic relationship. This is also because the provision of partner support fulfills fundamental human needs for love, comfort, and belongingness (Cutrona & Russell, 2017; Gillath et al., 2016) which can mitigate feelings of loneliness that can ensue when there is lack of an intimate attachment to a close other such as a romantic partner (Cutrona & Russell, 2017). Perceiving a partner as providing support is likely to be especially important during the pandemic where social restrictions, such as stay-at-home orders, disrupt the ecology of families such that parents are significantly limited in their capacity to draw upon other members of their social network and community, who are typically relied upon for instrumental and emotional support (e.g., Cochran, & Niego, 2002).

## The Current Study

Our study had two broad research aims. The first was to determine whether different profiles of enduring personal vulnerabilities (psychological distress and attachment insecurity), life stressors (pre-pandemic and COVID-19 stressors), and adaptive relationship processes (constructive communication and partner support) were associated with different trajectories of parents’ relationship satisfaction and loneliness. The second was to investigate whether the trajectories of relationship satisfaction and loneliness evidenced across different profile groups varied as a function of differences in lockdown orders between Victorians and non-Victorians.

## Method

### Participants

The current study drew on longitudinal data from Australian parents in a romantic relationship recruited as part of the COVID-19 Pandemic Adjustment Survey (CPAS; see study protocol, Westrupp et al., 2020) (*N* = 1942; 1476 women, 427 men, 39 gender not specified; *M* age = 38.27 years, *SD* = 6.88). On average, participating parents had two children living at home. The majority of parents had a university-level education (i.e., bachelor’s degree or higher; *n* = 1,305); otherwise, participants reported they had completed a high school certificate (*n* = 256); had obtained a trade certificate, diploma, or apprenticeship (*n* = 388); or had not completed high school (*n* = 141). The majority of the sample reported moderate or high household income, with only 14% reporting low household income (< $52,000 per annum). Seventy-six percent of participants reported they were in paid employment prior to the COVID-19 pandemic, whereas 17% had been unemployed and 7% did not report their employment status.

### Materials and Procedures

The study was approved by Deakin University’s Human Research Ethics Committee. Full details regarding the study are described in Westrupp et al. (2020). Parents were recruited via paid and unpaid social media advertisements containing a hyperlink directing parents to a study website in which they read through a plain language statement, provided online consent, and then undertook the initial survey (i.e., baseline [T1]) via Qualtrics. The baseline (T1) data was collected during April 2020 when all Australian states had entered a “level 3” national lockdown. Beyond the collection of demographic information, the baseline survey included measures of relationship satisfaction and loneliness as well as enduring personal vulnerabilities (psychological distress and attachment insecurity), adaptive relationship processes (constructive communication strategies and partner support), and pre-pandemic stress and COVID-19 stressors. The baseline survey took participants approximately 40 min to complete.

Following the baseline (T1) survey, participants completed measures of relationship satisfaction and loneliness every 2 to 4 weeks across the follow-up period (T2–T14) spanning 13.5 months. Our multiple follow-up waves were designed to capture the changing social restrictions over the course of the pandemic in Australia but planned for intermittent participation across multiple timepoints. The follow-up assessments took participants approximately 10 min to complete. From timepoint four, we conducted phone or text/SMS follow-up only if participants had not completed 3 + consecutive timepoints. Our approach was guided by the strengths of our trajectory modelling approach, which is robust to missing data and utilizes all available data from each participant (e.g., even if individuals participate at just 2–3 timepoints). Additionally, our approach maximized coverage over time, with a proportional spread of data over the timepoints of the study. Specifically, 72% of our final sample participated in three or more timepoints; 60% participated in 4 or more; and 62% participated during the second-wave Victorian lock-down (see Online Resource 1 for full details of data coverage across the study).

Throughout the follow-up period, government responses regarding social restrictions at the state-level within Australia differed greatly due to varied success in suppressing the spread of the COVID-19 outbreak. By late May 2020 (T4), all Australian states and territories had eased COVID-19-related restrictions. However, by the end of June 2020 (T6), level 4 lockdown restrictions were introduced in the state of Victoria. Victorian residents were subjected to these restrictions until late October (T10) after which time lockdown restrictions were lifted until a snap 5-day lockdown was activated in mid-February 2021 (in between T13 and T14 follow-up assessments). In the sections that follow, we describe all measures.

#### Relationship Satisfaction

Relationship satisfaction was measured by one item from the Perceived Relationships Quality Component (PRQC) Questionnaire (Fletcher et al., 2000) on which participants are asked to rate the extent to which they are currently satisfied with their romantic relationship rated on a 7-point scale from 1 (*not at all*) to 7 (*extremely*).

#### Loneliness

Loneliness was assessed using 6-items from UCLA Loneliness Scale (Russell et al., 1980). Items are rated on a 4-point scale from 0 (*never*) to 4 (*always*). Items are summed to range between 0 and 24 with higher scores indicative of higher loneliness (α = .82).

#### Personal Vulnerabilities

##### Psychological Distress

The short version of the Depression, Anxiety, and Scale Stress (DASS-21; Lovibond & Lovibond, 1995) was used to assess symptoms of depression, anxiety, and stress symptom. Each symptom type is assessed using 7-items rated on a 4-point scale from 0 (*did not apply to me at all*) to 3 (*applied to me very much, or most of the time*). Items on each subscale are summed and an overall score for psychological distress is derived by averaging across all subscales with higher scores reflecting greater psychological distress (α = .93).

##### Attachment Insecurity

Attachment insecurity towards a romantic partner was measured using the romantic partner items of Experiences in Close Relationships Scale-Relationship Structures (ECR-RS; Fraley et al., 2011). The measure consists of two subscales assessing the two primary dimensions that underpin attachment insecurity: attachment anxiety (3 items; α = .90) and attachment avoidance (6 items; α = .86). Items are rated on a 7-point scale from 1 (*strongly disagree*) to 7 (*strongly agree*) with higher scores on each subscale reflecting higher attachment insecurity.

#### Relationship Adaptive Processes

##### Constructive Communication

The adapted short-form of the Self-Expressiveness in the Family Questionnaire (Haberstadt et al., 1995) was used to assess participants positive (5-items; α = .87) and negative (6 items; α = .90) verbal and non-verbal communication within their family. All items are rated on a 9-point scale from 1 (*not at all frequently in my family*) to 9 (*very frequently in my family*). Higher scores on each scale represent either positive or negative self-expressiveness, respectively.

##### Perceived Partner Support

Perceived partner support was assessed using two items adapted for the current study based on established measures of partner support (e.g., Cutrona & Russell, 2017; Feeney & Thrush, 2010). One item tapped into the perception of esteem support (i.e., encouragement, affirmation of skills and abilities) and comfort support (i.e., validation, comfort and understanding) provided by one’s partner. Both items were rated on 7-point scale from (*strongly disagre*e) to 7 (*strongly agree*). Items were averaged such that higher scores indicate greater perceive partner support (α = .87).

#### Stressors

##### Pre-pandemic Stress

Pre-existing stressors were assessed at baseline using an adapted version of the Life Events Survey (Brugha & Cragg, 1990). Participants indicated whether they had experienced a total of 8 stressful events over the last 12 months prior to the COVID-19 pandemic on a dichotomous response format (yes/no). Items included stressors such as financial difficulties, injury or illness, job loss, and relationship difficulties. Items were summed such that higher scores reflected a greater number of pre-pandemic stressors.

##### COVID-19 Stress

Participants were asked to indicate whether they had experienced a total of seven stressors during the COVID-19 pandemic on a dichotomous response format (yes/no). Items were summed to form a count variable (range, 0–7) and included the following stressors: housing insecurity, financial insecurity; job loss, new job, reduction or increases in work hours, or changes (“redeployment”) in employment; food shortages; and COVID-19 illness (contracting the COVID-19 virus, hospitalization of themselves or a family member due to infection, and death of a family member due to the virus).

### Data Analysis

All data analyses were conducted in Mplus version 8.4 (Muthén & Muthén, 2017) using full information maximum likelihood (FIML) estimation. Analyses were conducted in three stages. First, unconditional latent growth curve models (LGCMs) were estimated separately for relationship satisfaction and loneliness. Each LGCM was estimated for the entire sample to determine the trajectory that best fit the data for relationship satisfaction and loneliness over time. Time was centered at 100 days (sample mean; note, baseline = time -100 days) to minimize convergence issues due to collinearity among growth trajectory components (e.g., linear, quadratic, cubic). Each of the unconditional LGCMs included an intercept as well as the modelling of linear, quadratic, cubic, and quartic growth parameters to capture linear and non-linear change in trajectories over the study period. The intercept reflects time-centered relationship satisfaction and loneliness, while the slope determines the trajectory of change for each outcome. To compare the fit of each model trajectory and determine the optimal growth parameter for each outcome, the loglikelihood chi-square values of each model trajectory was estimated and chi-square difference tests (Δχ^2^) of model fit were performed alongside inspection of key goodness-of-fit indices including Akaike information criterion (AIC) and Bayesian information criterion (BIC) values. The model that was the most parsimonious and demonstrated the best fit to the observed data of the full sample across the loglikelihood estimates, AIC and BIC indices was used for all subsequent analyses for relationship satisfaction and loneliness respectively.

Second, exploratory latent profile analyses (LPAs) were conducted to identify distinct profiles of parents based on baseline assessments of enduring personal vulnerabilities (psychological distress and attachment insecurity), life stress (COVID-19 stress and pre-pandemic stress), and adaptive relationship processes (constructive communication strategies and perceived partner support). A series of LPA models were run to examine solutions with increasing numbers of-classes (from 2 to 4), using a profile-invariant diagonal variance–covariance matrix (Mplus default) which constrains the variances of each measure to be equal across classes (Muthen & Muthen, 2017). The optimal latent profile-solution was based on several goodness-of-fit indices, including Vuong-Lo-Mendell-Rubin (VLMR) and Lo-Mendell-Rubin (LMR) likelihood ratio test *p* values, and lower AIC and BIC values. The optimal latent profile-solution was also based on entropy values, parsimony, and theoretical justification for interpreting latent profiles (Geiser, 2012). The most likely profile membership was also verified by high average profile assignment probability (> .80; Rost, 2006). Thus, selection of the optimal latent profile solution was based on the extent that a given solution demonstrated improved fit across all outlined goodness-of-fit-indices while also reflecting a parsimonious and theoretically justifiable solution.

Third, conditional LGCMs, using latent profile membership as covariates, were estimated for relationship satisfaction and loneliness separately. Analyses included a grouping variable to distinguish between Victorian and non-Victorian participants so that differences between Victorian and non-Victorian trajectories could be tested across the identified latent profiles. Wald test of parameter constraints were conducted to test for differences in the intercept and growth components of the estimated trajectories for Victorian and non-Victorian participants. Two sets of Wald tests were conducted. The first tested whether all components of the relationship satisfaction and loneliness trajectories differed across latent profiles *within* Victorian participants and *within* non-Victorian participants. The second tested for whether the trajectories for relationship satisfaction and loneliness differed *between* Victorian and non-Victorian participants for each latent profile.

## Results

### Unconditional LGCMs: Determining Relationship Well-Being Trajectories for the Entire Sample

**Fig. 1:**
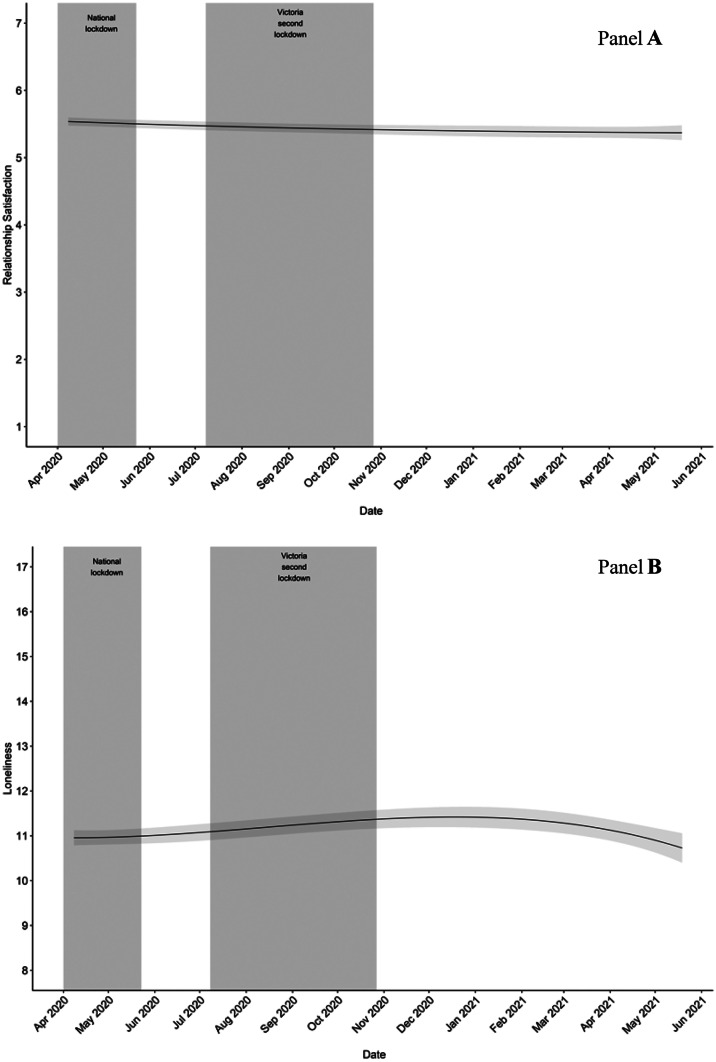
Unconditional latent growth trajectories for parent relationship satisfaction and loneliness. Note. Gray shadow indicates 95% lower bound and upper bound confidence intervals

Fit indices, mean, and variance estimates of the growth parameters (i.e., linear, quadratic, cubic and quartic) for relationship satisfaction and loneliness are presented in Table 1. Although the chi-square likelihood and AIC values suggested a quartic trajectory to best fit the data for both outcomes, change in BIC values across estimated trajectories indicated a cubic trajectory was of best fit for both relationship satisfaction and loneliness. Given that our decision for choosing a trajectory was based on model parsimony and that all fit indices demonstrated improvement (relative to alternative tested trajectories; see data analysis), we decided that the cubic trajectory was the most appropriate fit to the data. The estimated mean cubic trajectory for each outcome is illustrated in Fig. 1 (see panels a and b). As shown in Fig. 1 (panel a), relationship satisfaction demonstrated a decline across the full study period. As shown in Fig. 1 (panel b), loneliness demonstrated an increase during lockdown restrictions, followed by a reduction in loneliness as lockdown restrictions were released.

**Table 1:** Unconditional LGCM model fit statistics for relationship quality

**Model**	**df**	**Loglikelihood**	**Loglikelihood scaling** **correction factor for MLR**	**AIC**	**BIC**	Δ χ^2^ **Test**	Δ **AIC**	Δ **BIC**
**Relationship quality**								
Linear	6	−13127.49	2.25	26266.98	26310.48			
Quadratic	10	−13083.85	2.03	26187.69	26260.18	Δ χ2(4) = 51.79, *p* < 0.001	79.29	50.30
Cubic	15	−13052.38	1.89	26134.75	26243.48	Δ χ2(5) = 39.10, *p* < 0.001	52.94	16.69
Quartic	21	−13040.61	1.62	26123.22	26275.44	Δ χ2(6) = 25.13, *p* < 0.001	11.54	-31.95
**Loneliness**								
Linear	6	−24536.92	1.59	49085.85	49129.37			
Quadratic	10	−24435.27	1.50	48890.54	48963.08	Δ χ2(4) = 149.63, *p* < 0.001	195.31	166.29
Cubic	15	−24395.34	1.41	48820.69	48929.49	Δ χ2(5) = 65.41, *p* < 0.001	69.85	33.58
Quartic	21	−24369.54	1.42	48781.07	48933.41	Δ χ2(6) = 35.41, *p* < 0.001	39.61	-3.91

Δ χ^2^ test and change columns denote a change in statistics from previous model (e.g., quadratic Δ χ^2^ test and change indicate difference between quadratic model and linear model)

### Latent Profile Analysis (LPA): Establishing Profiles of Vulnerabilities, Stress, and Adaptations

Fit statistics for the LPA are presented in Table 2. The LMR and VLMR *p* values suggested that a 3-profile solution was of fit better than a 2-profile solution, which was of better fit than a 1-profile solution. This was also reflected in the reduction in the AIC and BIC values across the estimation of a 1- to 3-profile solution. It is important to note, however, that although the AIC and BIC values also decline from the 3-profile to 4-profile solution, the 4-profile solution did not show better fit based on the LMR and VLMR *p* values, nor was there an improvement in entropy. Given that the 4-profile solution did not demonstrate a consistent improvement in fit across all indices and entropy, it was determined that a 3-profile solution was best representative of the sample and reflective of a more parsimonious solution.

**Table 2:** Class fits for LPA

**Profiles (k)**	**AIC**	**BIC**	**VLMR** ***p*-value**	**LMR** ***p*-value**	**Entropy**	**Profile**	***n***	**%**
1								
2	54391.27	54530.56	< 0.001	< 0.001	0.72	1	1364	70.2
						2	578	29.8
3	53956.30	54145.74	0.037	0.039	0.77	1	336	17.3
						2	1299	66.9
						3	307	15.8
4	53676.75	53916.32	0.427	0.431	0.77	1	323	16.6
						2	364	18.7
						3	1144	58.9
						4	111	5.7

Acceptable to high profile assignment probability was obtained for each of the three latent profiles constituting the 3-profile solution [Profile 1 [.96], Profile 2[.81], and Profile 3 [.78]). In terms of the percentage of participants assigned to each profile, 66.9% were in Profile 1 (*n* = 1299), 17.3% were in the Profile 2 (*n* = 336), and 15.8% were in Profile 3 (*n* = 307) (see Table 2).

Additionally, the 3-profile solution demonstrated a similar sample distribution across Victorian and non-Victorian participant groups (P1: *n*_[Victorian]_ = 647,* n*_[non-Victorian]_ = 652; P2: *n*_[Victorian]_ = 164, *n*_[non- Victorian]_ = 172; P3: *n*_[Victorian]_ = 162, *n*_[non- Victorian]_ = 145). The means, standard errors, and scale ranges for the enduring personal vulnerabilities, adaptive relationship processes, and life stressors as assessed at baseline for the 3-profile solution are presented in Table 3. Given that the three profiles demonstrate some similarities and important points of difference, we decided to label the profiles in a parsimonious way that best captured these similarities and points of difference.

**Table 3:** Means and standard error for personal vulnerabilities, relationship adaptations, pre-pandemic stress, and COVID-19-related stress subscales for each of the three latent profiles (*N* = 1942)

		**Scale** **Range**	**Profile 1** **[Low vulnerabilities/high adaptations]** **(reference)**		**Profile 2** **[Moderate vulnerabilities/adaptations]**		**Profile 3** **[High vulnerabilities/adaptations]**	
			***M***	**SE**	***M***	**SE**	***M***	**SE**
**Personal vulnerabilities**	Attachment avoidance	1–7	2.92	0.05	3.92	0.11	3.98	0.11
	Attachment anxiety	1–7	2.51	0.11	3.88	0.17	5.16	0.15
	Psychological distress	0–21	3.66	0.09	5.95	0.40	9.35	1.14
**Relationship adaptations**	Partner support	1–7	5.92	0.08	2.95	0.19	5.25	0.19
	Negative expression	1–9	3.27	0.07	5.23	0.16	4.78	0.16
	Positive expression	1–9	7.54	0.04	5.47	0.24	7.13	0.16
**Life stressors**	Pre-pandemic stress	0–8	.71	0.03	1.28	0.09	1.40	0.12
	COVID-19 stress	0–7	1.16	0.04	1.55	0.09	2.01	0.17

As can be seen from Table 3, the profile of mean scores for participants classified into Profile 1 (P1) reflected low enduring personal vulnerabilities (low attachment insecurity and psychological distress), high relationship adaptations (high perceived partner support and constructive communication [high positive expressiveness and low negative expressiveness]), and low stress (pre-pandemic and COVID-19). Given this profile, we labelled P1 as the “low vulnerabilities/high adaptations” group. The profile of mean scores for participants classified into Profile 2 (P2) reflected moderate enduring personal vulnerabilities (moderate attachment insecurity, moderately low psychological distress^1^), moderate relationship adaptations (moderately low partner support and moderate levels of constructive communication [moderate positive and negative expressiveness]), and low stress. Given this profile, we labelled P2 the “moderate vulnerabilities/adaptations” group. Finally, the profile of mean scores for Profile 3 (P3) reflected high vulnerabilities (moderate to high attachment insecurity, high psychological distress), high relationship adaptations (high partner support and constructive communication (high positive expressiveness and moderate negative expressiveness), and low stress. Given this profile, we labelled P3 the “high vulnerabilities/adaptations” group.

Participants in the moderate vulnerabilities/adaptations group (P2) scored the lowest on baseline measures of relationship adaptations compared to participants in the other profiles. Additionally, participants in the moderate vulnerabilities/adaptations group (P2) scored higher on baseline measures of personal vulnerabilities, pre-pandemic stress, and baseline measures of COVID-19-related stress than the low vulnerabilities/high adaptations group (P1), but lower than participants in the high vulnerabilities/adaptations group (P3). Participants in the high vulnerabilities/adaptations group (P3) scored highest across baseline measures of personal vulnerabilities, pre-pandemic stress and COVID-19-related stress than other profiles. Additionally, participants in the high vulnerabilities/adaptations group (P3) had lower scores on baseline measures of relationship adaptations than the participants in the low vulnerabilities/high adaptations group (P1), but higher scores than those in the moderate vulnerabilities/adaptations group (P2).

### Conditional LGCM: Trajectories of Relationship Well-Being as a Function of VSAM Profiles and Lockdown Policies

**Fig. 2:**
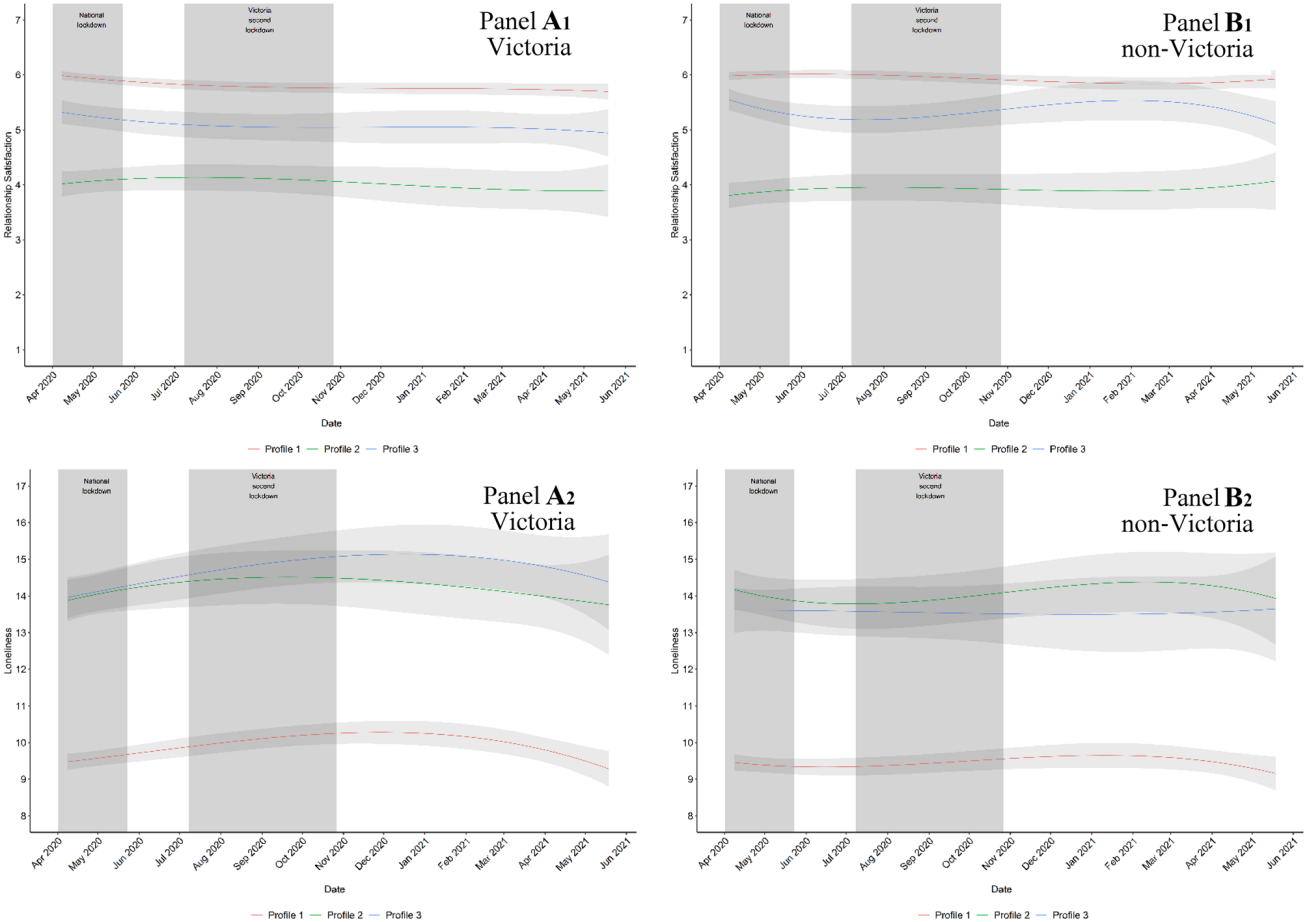
Relationship satisfaction and loneliness trajectories for latent profiles grouped by Victorian and non-Victorian parents. Note. Profile 1 = low vulnerabilities/high adaptations; Profile 2 = moderate vulnerabilities/adaptations; Profile 3 = high vulnerabilities/adaptations. Gray shadow indicates 95% lower bound and upper bound confidence intervals

Conditional LGCMS were estimated in which the cubic trajectory for relationship satisfaction and loneliness was modelled with latent profile membership as a covariate and participants’ state of residence (Victorian vs non-Victorian) as a grouping variable. For both relationship satisfaction and loneliness, the trajectories were calculated separately for all three latent profiles for Victorian and non-Victorian participants. This resulted in the estimation of six trajectories for relationship satisfaction and six trajectories for loneliness. These trajectories are illustrated in Fig. 2 and differences are presented in Table 4.

**Table 4:** Significance test for comparisons of growth parameters within Victorian and Non-Victorian profile groups (*p* value for Wald test of parameter constraints)

			Overall	Intercept	Linear	Quadratic	Cubic
**Relationship satisfaction**							
*Within* Vic group							
	Profile 1	Profile 2	< 0.001	< 0.001	0.180	0.120	0.338
		Profile 3	< 0.001	< 0.001	0.854	0.681	0.748
	Profile 2	Profile 3	< 0.001	< 0.001	0.261	0.136	0.347
*Within* non-Vic group							
	Profile 1	Profile 2	< 0.001	< 0.001	0.270	0.705	0.794
		Profile 3	< 0.001	< 0.001	0.543	0.001	0.002
	Profile 2	Profile 3	< 0.001	< 0.001	0.769	0.004	0.012
*Between* Vic and non-Vic groups							
	Profile 1 Vic	Profile 1 Non-Vic	0.004	0.002	0.156	0.004	0.026
	Profile 2 Vic	Profile 2 Non-Vic	0.548	0.291	0.809	0.971	0.866
	Profile 3 Vic	Profile 3 Non-Vic	0.323	0.556	0.300	0.166	0.173

**Loneliness**							
*Within* Vic group							
	Profile 1	Profile 2	< 0.001	< 0.001	0.450	0.512	0.472
		Profile 3	< 0.001	< 0.001	0.439	0.832	0.882
	Profile 2	Profile 3	0.626	0.676	0.247	0.708	0.645
*Within* non-Vic group							
	Profile 1	Profile 2	< 0.001	< 0.001	0.792	0.430	0.605
		Profile 3	< 0.001	< 0.001	0.476	0.571	0.470
	Profile 2	Profile 3	0.638	0.670	0.652	0.320	0.349
*Between* Vic and non-Vic groups							
	Profile 1 Vic	Profile 1 non-Vic	0.002	0.002	0.002	0.107	0.686
	Profile 2 Vic	Profile 2 non-Vic	0.288	0.203	0.393	0.087	0.269
	Profile 3 Vic	Profile 3 non-Vic	0.073	0.027	0.031	0.779	0.729

Profile 1 = low vulnerabilities/high adaptations, Profile 2 = moderate vulnerabilities/adaptations, Profile 3 = high vulnerabilities/adaptations; Vic = Victorian

#### Trajectory Differences Within Victorian and Non-Victorian Participants

Wald test of parameter constraints was conducted to test for significant differences in the trajectories of relationship satisfaction and loneliness between each profile *within* Victorian participants and *within* non-Victorian participants.

##### Relationship Satisfaction

Tests revealed that the trajectory differed between all profiles for Victorian participants.However, profiles significantly differed at the intercept only (see Online Resource 2, Table 4, and Fig. 2 panel A1). Participants in the low vulnerabilities/high adaptations group (P1) demonstrated the highest relationship satisfaction across the entire period of changing social restrictions compared to the other two profile groups. Participants in the moderate vulnerabilities/adaptation group (P2) demonstrated the lowest relationship satisfaction across time, whereas participants in the high vulnerabilities/adaptations profile group (P3) evidenced levels of relationship satisfaction that fell between those of the other two profile groups. For the non-Victorian participants, all profiles had significantly different trajectories from one another (see Table 4 and Fig. 2 panel B1). In terms of differences in specific growth parameters, all profiles differed at the intercept; however, participants in the high vulnerabilities/adaptations group (P3) also differed from participants in the other two profiles across the quadratic and cubic growth components of the trajectory for relationship satisfaction. Specifically, for the non-Victorian participants, those in the high vulnerabilities/adaptions group (P3) demonstrated significantly greater fluctuation in satisfaction. In contrast, the other two profiles demonstrated similar trajectories over the study period, the only difference being their overall levels of relationship satisfaction.

##### Loneliness

In comparison to the low vulnerabilities/high adaptations group (P1), both the moderate vulnerabilities/adaptation group (P2) and high vulnerabilities/adaptations profile group (P3) demonstrated significant differences in loneliness trajectories for Victorian participants (see Online Resource 2, Table 4, Fig. 2 panel A2). However, tests across all growth components revealed that the profiles all differed from one another at the intercept only. Specifically, participants in the low vulnerabilities/high relationship adaptations group (P1) demonstrated the lowest loneliness across the entire period of changing social restrictions compared to the other two profile groups. The same results were found for non-Victorian participants, with differences between the profiles only evidenced for the intercept. The low vulnerabilities/high adaptations group (P1) demonstrated the lowest loneliness over time.

#### Trajectory Differences Between Victorian and Non-Victorian Participants

Wald tests were conducted to determine whether the trajectories for each profile differed between Victorian and non-Victorian participants for relationship satisfaction and loneliness.

##### Relationship Satisfaction

 The trajectory for relationship satisfaction differed between Victorian and non-Victorian participants for the low vulnerabilities/high adaptations group (P1), but not between Victorian and non-Victorian participants clustered in the other profiles (see Table 4). In terms of the low vulnerabilities/high adaptations group (P1), Victorian and non-Victorian participants differed at the intercept, quadratic, and cubic growth components. As shown in Fig. 2 (comparing panels A1 and B1), the Victorian participants in the low vulnerabilities/high adaptations group (P1) experienced sharper declines in relationship satisfaction during the second Victorian lockdown period compared to non-Victorian participants from the same latent profile group. For the other two profile groups, Victorian and non-Victorian participants experienced similar trajectories of relationship satisfaction.

##### Loneliness

In terms of loneliness, both the low vulnerabilities/high adaptations group (P1) and high vulnerabilities/adaptations profile group (P3) demonstrated differences between Victorian and non-Victorian participants across latent profiles. For both Victorian and non-Victorian participants, these latent profile groups demonstrated differences at the intercept and linear components of the loneliness growth trajectory. As shown in Fig. 2 (comparing panels A2 and B2), participants from Victoria in either the low vulnerabilities/high adaptations group (P1) or high vulnerabilities/adaptations profile group (P3) demonstrated higher levels of loneliness throughout the entire period of changing lockdown restrictions, and sharper linear increases that peaked at approximately two-thirds of the way during the second Victorian lockdown compared to non-Victorian participants. For the moderate vulnerabilities/adaptation group (P2), Victorian and non-Victorian participants experienced similar trajectories of loneliness.

## Discussion

Understanding the relational toll of strict lockdowns on parents is particularly important given the increased workload and responsibilities that parents have shouldered while being isolated from their broader social network (Evans et al., 2020; McCrae et al., 2021). These challenges have likely compromised the time and effort that many parents can devote to maintaining satisfying relationships (McCrae et al., 2021; Westrupp et al., 2022a, b). Despite this concern, there exists little research exploring the relationship well-being (i.e., relationship satisfaction and loneliness) of parents over the course of the pandemic. In addressing this gap, we drew on a large sample of Australian parents that were assessed over a 14-month period during which time parts of the nation experienced different stay-at home-orders in terms of length and strictness (Victoria versus the rest of Australia). These differences provided a unique natural experiment to assess the effects of strict lockdowns on relationship well-being. However, in acknowledging that not all parents will experience the same changes in relationship well-being over the course of changing social restrictions, we situated the study of stay-at-home orders within the Vulnerability Stress Adaptation Model (VSAM, Karney & Bradbury, 1995). Theoretical applications of the VSAM to the COVID-19 pandemic highlight the critical role that enduing personal vulnerabilities, life stressors, and adaptive relationship processes are likely to play in understanding the trajectory of relationship well-being during the pandemic (Karantzas et al., 2022; Karney & Bradbury, 1995; Pietromonaco & Overall, 2021).

Our study had two broad research aims. The first was to determine whether different profiles of enduring personal vulnerabilities, life stressors, and adaptive relationship processes were associated with different trajectories of parents’ relationship satisfaction and loneliness. The second was to investigate whether the trajectories of relationship satisfaction and loneliness evidenced across different profile groups of parents varied as a function of differences in lockdown orders between Victorian and non-Victorian citizens.

In relation to our first aim, we identified three latent profiles on the basis of baseline levels of personal vulnerabilities, adaptive relationship processes, and life stressors. These profiles all demonstrated low levels of pre-pandemic and COVID-19 stressors; however, the three profiles varied in terms of personal vulnerabilities and adaptive relationship processes. These three profiles were labeled as parents with: (1) low vulnerabilities/high adaptations, (2) moderate vulnerabilities/adaptations, and (3) high vulnerabilities/adaptations. All profiles were found to demonstrate cubic non-linear changes in relationship satisfaction and loneliness. However, parents in the low vulnerabilities/high adaptations group experienced relatively high levels of relationship satisfaction and low levels of loneliness over the first 14 months of the pandemic compared to the other two latent profile groups. Furthermore, parents in the high vulnerabilities/adaptations group demonstrated higher relationship satisfaction and less loneliness than parents who evidenced moderate vulnerabilities/adaptations.

These findings provide support for the VSAM which propose that relationship wellbeing is largely determined by a complex interplay between levels of personal vulnerabilities, life stressors, and adaptive relationship processes (Karantzas et al., 2022; Karney & Bradbury, 1995; Pietromonaco & Overall, 2021, 2022). Indeed, our findings highlight that parents who demonstrate different profiles of personal vulnerabilities and adaptive relationship processes in the early stages of the pandemic experience different levels of relationship satisfaction and loneliness throughout the pandemic and evolving social restrictions. However, our findings also highlight the particular importance of adaptive relationship processes in ensuring that parents can maintain moderate to high relationship satisfaction and to mitigate feelings of loneliness over the course of the COVID-19 social restrictions.

The findings speak to the critical importance of parents’ abilities to enact adaptive relationship processes in a bid to maintain relationship well-being. Specifically, the ability for parents to constructively communicate with one another (through enacting positive self-expression and inhibiting negative self-expression) and to perceive relationship partners as supportive are critical in strengthening romantic bonds, especially during times of crisis as in the current pandemic. In the context of COVID-19, the use of constructive communication by parents is important because it enables the clear communication of needs, fears, and apprehensions as to how parents can manage responsibilities and virus concerns as well as help each other maintain a sense of emotional and relational well-being during strict social restrictions (Karantzas et al., 2022; Pietromonaco & Overall, 2021; Overall et al., 2022). Furthermore, perceiving support from a romantic partner that is sensitive and responsive has both a stress-buffering effect in times of crisis and enhances belongingness, relationship closeness, and satisfaction because the romantic partner is acknowledged as effectively attending to another’s needs (Feeney & Thrush, 2010; Gillath et al., 2016). This, in turn, promotes love, validation, and understanding (Cutrona & Russell, 1990, 2017; Reis & Clark, 2013). Moreover, during strict social restrictions, perceiving the support of a partner is likely to be especially important to parents in the maintenance of relational wellbeing because of they are less able to draw upon their usual network of social ties for assistance with child responsibilities and to receive emotional and instrumental help (e.g., Cochran, & Niego, 2002).

In terms of our second aim, differences in the mandating of strict lockdowns were evidenced in the relationship satisfaction and loneliness experienced by Victorian and non-Victorian participants. Specifically, differences were found between Victorian and non-Victorian participants, but only for those parents in profile groups evidencing high relationship adaptations. This is a surprising finding in that latent profile groups with high relationship adaptations were those who reported differences in relationship well-being as a functioning of divergent lockdown experiences. In terms of relationship satisfaction, Victorian parents evidencing low vulnerabilities and high relationship adaptations experienced sharper declines in relationship satisfaction during lockdown periods (i.e., the national lockdown and Victorian lockdown) compared to non-Victorian parents. Furthermore, Victorian parents in profile groups evidencing high adaptations (i.e., P1, low vulnerabilities/high adaptations and P3, high vulnerabilities/ adaptations) demonstrated higher levels of loneliness throughout the entire period of changing lockdown restrictions. Furthermore, in Victorian parents, these profile groups demonstrated sharper linear increases and decreases that peaked approximately two-thirds during the second Victorian lockdown compared to non-Victorian parents.

It may well be that parents who report high relationship adaptations (and little by way of vulnerabilities) experience quite high relationship well-being and, therefore, have more room to experience declines in relationship satisfaction and increases in loneliness. These high levels of satisfaction and low loneliness requires that people are able to effectively work on their relationships and maintain effort and attention (Ogolsky & Monk, 2019). Therefore, it may be that when strict lockdowns are enforced on multiple occasions that the ability of parents to a maintain high level of relationship well-being is indeed compromised as increased responsibilities are sustained for extended periods of time. Furthermore, as strict lockdowns are re-instituted or continue to be enforced, parents are likely to be cut off from their extended social network and family ties for a long period of time—this isolation from the broader network may further exacerbate declines in satisfaction and a heightened sense of loneliness. Our findings suggest that strict social restrictions do appear to disrupt aspects of the human ecosystem, and indeed, these social disruptions can affect those who are assumed to be of least risk to experience significant changes in relational well-being.

## Implications

These findings have a number of important implications in terms of how government and service providers can best support the relational well-being of parents during the government institution of strict lockdowns. First, the non-linear trajectories of relationship satisfaction and loneliness evidenced throughout changing social restrictions suggest it is important to monitor relationship well-being of parents over time. Second, assessing the personal vulnerabilities, life stressors, and adaptive relationship processes of parents in the early stages of strict lockdown can identify profiles of parents who are especially likely to experience compromised relationship wellbeing throughout changing social restrictions. Instituting the assessment of personal vulnerabilities, life stressors, and adaptive processes in the initial stages of lockdown and the regular assessment of relationship well-being can identify parents who are most at risk of reductions in relationship satisfaction and increases in loneliness and periods during which downturns in relationship well-being are most likely to occur. Through leveraging digital technologies, parents can be administered such assessments on mobile devices to inform the timing of public health interventions to address the anticipated reductions in the relationship well-being of parents.

Third, our findings highlight that it is the absence of adaptive relationship processes, rather than the presence of personal vulnerabilities, that may be especially important in maintaining relationship well-being. As a case in point, parents who had high personal vulnerabilities but also had similar levels of relationship adaptations fared better over time in terms of relationship well-being compared to those with moderate personal vulnerabilities and adaptive relationship processes. Thus, providing parents with relationship education and counselling during times of social restrictions is critically important. In the context of social distancing guidelines, the delivery of relationship education or therapy requires the use of online technologies and implementation of digital innovations. Two such examples are the ePREP (Braithwaite & Fincham, 2007) and the OurRelationship (Doss et al, 2016) programs. Both programs are adaptations of established interventions (Prevention and Relationship Enhancement Program or PREP; Markman et al., 1993; and Integrative Behavioral Couple Therapy; Christensen & Doss, 2017). Furthermore, research indicates that both online programs can enhance adaptive relationship processes and relationship well-being (e.g., Braithwaite & Fincham, 2014; Doss & Rhoades, 2017). Importantly, such programs can be provided at a modest cost and are tailored to incorporate self-directed activities with telephone-based coaching support. These programs can also be of value to parents who already experience a high level of relationship well-being and wish to maintain such levels. Indeed, our comparison of strict lockdown regulations for Victorian and non-Victorian parents revealed that, Victorian parents with high relationship adaptations experienced decreased satisfaction and increased loneliness compared to non-Victorian parents. These differences were largely evidenced during the second strict lockdown experienced only by Victorian parents. Thus, it is important to make such programs available and accessible during multiple waves of strict social restrictions, even to those parents who typically report highly adaptive relationship functioning.

### Limitations and Future Directions

Although our findings provide novel and important insights into the relationship wellbeing of parents during changing COVID-19 social restrictions, there are some limitations that need to be noted. First, the identified latent profile groups all indexed low levels of pre-pandemic and COVID-19 stressors and none of the profiles included parents who evidenced very high personal vulnerabilities. Thus, we are unable to determine the extent to which very high levels of personal vulnerabilities and stress—which are known to negatively affect relationship wellbeing (Karney & Bradbury, 1995; Pietromonaco & Overall, 2021, 2022)—may be especially problematic during COVID-19 and periods of strict social restrictions.

Second, our data collection did not include pre-lockdown assessments of relationship well-being, enduring personal vulnerabilities, life stressors, and relationship adaptive processes. Thus, we are unable to comment on whether our measured constructs differed in levels prior to the activation of the national lockdown. However, there exists a large evidence-base to suggest that a number of the variables that comprised our latent profiles demonstrate greater stability than instability over time and contexts. These variables include attachment insecurity (Gillath et al., 2016), support processes (Cutrona & Russell, 2017), and communication patterns in couples (Christensen et al., 2020). In terms of the trajectories of relationship satisfaction and loneliness, the high number of repeated assessments during a 14-month period that included Victoria experiencing a second round of lockdown provides an opportunity to capture dynamics of relationship wellbeing for a subsample of the Australian population prior and post lockdown.

Third, despite attempts to recruit a large national representative sample of Australians, our sample predominantly consisted of parents with a high level of education and moderate to high socioeconomic status. Thus, our findings are limited in how they generalize to parents who experience significant social disadvantage, a factor which has been found in past research to have negative effects of relationship wellbeing (e.g., Maisel & Karney, 2012). Future research could specifically target parents who experience social disadvantage as well as those who experience mental health concerns and other enduring personal vulnerabilities. Doing so would help to determine the extent to which relationship wellbeing is especially compromised within these sub-populations during times of human ecological disruption brought about by strict social restrictions.

Fourth, our sample of parents consisted predominantly of women. Thus, our findings may not capture the relational experiences of fathers to the extent that they reflect the experiences of mothers. Having said this, models of relationship functioning tend to find little differences in associations between men and women regarding relationship processes (e.g., Karantzas et al., 2014; Kurdek, 2005). Indeed, emerging research examining gender differences in relationship wellbeing during the pandemic, generally suggests little by way of gender differences (e.g., Sels et al., 2022; Williamson, 2020). However, one study found that gender differences in relationship wellbeing emerged when women reported increases in household demands compared to men (Waddell et al., 2021). Future research can focus on targeting the greater inclusion of fathers, assessing the household and parenting demands of both mothers and fathers, as well as placing greater emphasis on the recruitment of dyads. The recruitment of parent dyads would also allow for the investigation of couple processes, such as how men’s latent profiles not only affect their own relationship wellbeing, but their partner’s relationship well-being, and vice-versa.

## Conclusion

Drawing on the VSAM, our paper reported on the effects of strict lockdown restrictions on the relationship well-being of Australian parents surveyed across the COVID-19 pandemic. Our findings provide novel insights into how government mandated lockdowns can disrupt the relational ecology of parents. Our findings highlight the importance of public health responses during the enforcement of strict lockdowns that provide parents with programs and resources to strengthen constructive communication and support to mitigate against declines in relationship satisfaction and increases in loneliness.

## Supplementary Information

Below is the link to the electronic supplementary material.

Supplementary file1 (DOCX 235 KB)

## Data Availability

On paper acceptance, data will be made available by application to the Australian Data Archive.

## Footnotes

1 Given that the sample of parents reflected a nation-wide community sample, determinations of low, moderate, and high mean levels of psychological distress were based on comparing mean level scores on the DASS-21 for each profile against population-based data reported in Crawford et al. (2011).

## References

[CR1] Beach, S. R., & Cassidy, J. F. (1991). The marital discord model of depression. Comprehensive Mental Health Care.

[CR2] Borkowska, M., & Laurence, J. (2021). Coming together or coming apart? Changes in social cohesion during the Covid-19 pandemic in England. *European Societies, 23(Supp)*, S618–S636.

[CR3] Bowlby, J. (1969/1982). *Attachment and loss, Vol. 1: Attachment*. Basic Books.

[CR4] Braithwaite S, Holt-Lunstad J (2017). Romantic relationships and mental health. Current Opinion in Psychology.

[CR5] Braithwaite, S. R., & Fincham, F. D. (2007). ePREP: Computer based prevention of relationship dysfunction, depression and anxiety. *Journal of Social and Clinical**Psychology*, *26*, 609–622.

[CR6] Braithwaite, S. R., & Fincham, F. D. (2014). Computer-based prevention of intimate partner violence in marriage. *Behaviour Research and Therapy*, *54*, 12–21. 10.1016/j.brat.2013.12.006 24463577

[CR7] Brennan KA, Clark CL, Shaver PR, Simpson JA, Rholes WS (1998). Self-report measurement of adult attachment: An integrative overview. Attachment theory and close relationships.

[CR8] Brugha TS, Cragg D (1990). The list of threatening experiences: The reliability and validity of a brief life events questionnaire. Acta Psychiatrica Scandinavica.

[CR9] Caporael LR, Brewer MB (1991). Reviving evolutionary psychology: Biology meets society. Journal of Social Issues.

[CR10] Christensen, A., & Doss, B. D. (2017). Integrative behavioral couple therapy. *Current Opinion in Psychology*, *13*, 111–114. 10.1016/j.copsyc.2016.04.022 PMC5096782 27822489

[CR11] Christensen, A., Doss, B. D., & Jacobson, N. S. (2020). Integrative Behavioral Couple Therapy: A therapist's guide to creating acceptance and change (2nd ed.). Norton.

[CR12] Cochran, M., & Niego, S. (2002). Parenting and social networks. *Handbook of parenting volume 4 social conditions and applied parenting*, 122.

[CR13] Crawford, J., Cayley, C., Lovibond, P. F., Wilson, P. H., & Hartley, C. (2011). Percentile norms and accompanying interval estimates from an Australian general adult population sample for self‐report mood scales (BAI, BDI, CRSD, CES‐D, DASS, DASS‐21, STAI‐X, STAI‐Y, SRDS, and SRAS). *Australian Psychologist*, *46*(1), 3–14.

[CR14] Cutrona CE, Russell DW (1987). The provisions of social relationships and adaptation to stress. Advances in Personal Relationships.

[CR15] Cutrona, C. E., & Russell, D. W. (1990). Type of social support and specific stress: Toward a theory of optimal matching. In B.R. Sarason, I. G. Sarason, & G.R. Pierce (Eds.), *Wiley series on personality processes. Social support: An interactional view* (p. 319–366). John Wiley & Sons.

[CR16] Cutrona CE, Russell DW (2017). Autonomy promotion, responsiveness, and emotion regulation promote effective social support in times of stress. Current Opinion in Psychology.

[CR17] Department of Health. (2020). Coronavirus (COVID-19) current situation and case numbers. Commonwealth of Australia Department of Health. Retrieved April 27, 2021, from https://www.health.gov.au/news/health-alerts/novel-coronavirus-2019-ncov-health-alert/coronavirus-covid-19-current-situation-and-case-numbers

[CR18] Doss BD, Rhoades GK (2017). The transition to parenthood: Impact on couples’ romantic relationships. Current Opinion in Psychology.

[CR19] Doss, B. D., Cicila, L. N., Georgia, E. J., Roddy, M. K., Nowlan, K. M., Benson, L. A., & Christensen, A. (2016). A randomized controlled trial of the web-based OurRelationship program: Effects on relationship and individual functioning. *Journal of Consulting and Clinical Psychology*, *84*(4), 285–296. 10.1037/ccp0000063 10.1037/ccp0000063 PMC4804631 26999504

[CR20] Evans S, Mikocka-Walus A, Klas A, Olive L, Sciberras E, Karantzas G, Westrupp EM (2020). From ‘It has stopped our lives’ to ‘Spending more time together has strengthened bonds': The varied experiences of Australian families during COVID-19. Frontiers in Psychology.

[CR21] Fancourt D, Steptoe A, Bu F (2021). Trajectories of anxiety and depressive symptoms during enforced isolation due to COVID-19 in England: A longitudinal observational study. The Lancet Psychiatry.

[CR22] Feeney BC, Thrush RL (2010). Relationship influences on exploration in adulthood: The characteristics and function of a secure base. Journal of Personality and Social Psychology.

[CR23] Feeney, J. A. (2016). Adult romantic attachment: Developments in the study of couple relationships. In J. Cassidy & P. R. Shaver (Eds.), *Handbook of attachment: Theory, research, and clinical applications* (3rd ed., p. 435–463). Guilford.

[CR24] Fletcher GJ, Simpson JA, Thomas G (2000). The measurement of perceived relationship quality components: A confirmatory factor analytic approach. Personality and Social Psychology Bulletin.

[CR25] Fraley RC, Heffernan ME, Vicary AM, Brumbaugh CC (2011). The experiences in close relationships—Relationship Structures Questionnaire: A method for assessing attachment orientations across relationships. Psychological Assessment.

[CR26] Fraley RC, Waller NG, Brennan KA (2000). An item response theory analysis of self-report measures of adult attachment. Journal of Personality and Social Psychology.

[CR27] Fredman SJ, Monson CM, Schumm JA, Adair KC, Taft CT, Resick PA (2010). Associations among disaster exposure, intimate relationship adjustment, and PTSD symptoms: Can disaster exposure enhance a relationship?. Journal of Traumatic Stress.

[CR28] Geiser, C. (2012). Data Analysis with Mplus. Guilford Publications.

[CR29] Gillath, O., Karantzas, G. C., & Fraley, R. C. (2016). *Adult attachment: A concise introduction to theory and research*. Academic Press.

[CR30] Halberstadt AG, Cassidy J, Stifter CA, Parke RD, Fox NA (1995). Self-expressiveness within the family context: Psychometric support for a new measure. Psychological Assessment.

[CR31] Hammen C (1991). Generation of stress in the course of unipolar depression. Journal of Abnormal Psychology.

[CR32] Karantzas GC, Feeney JA, Agnew CR, Christensen A, Cutrona CE, Simpson JA (2022). Dealing with loss in the face of disasters and crises: Integrating interpersonal theories of couple adaptation and functioning. Current Opinion in Psychology.

[CR33] Karantzas GC, Feeney JA, Wilkinson R (2010). Is less more? Confirmatory factor analysis of the Attachment Style Questionnaires. Journal of Social and Personal Relationships.

[CR34] Karantzas GC, Feeney JA, Goncalves CV, McCabe MP (2014). Towards an integrative attachment-based model of relationship functioning. British Journal of Psychology.

[CR35] Karney BR, Bradbury TN (1995). The longitudinal course of marital quality and stability: A review of theory, methods, and research. Psychological Bulletin.

[CR36] Knox, L., Karantzas, G. C., Romano, D., Feeney, J. A., & Simpson, J. A. (2022). One Year On: What we have learned about the psychological effects of Covid-19 social restrictions–a meta-analysis. *Current Opinion in Psychology*, 101315. 10.1016/j.copsyc.2022.101315 PMC8907153 35398753

[CR37] Kurdek L (2005). Gender and marital satisfaction early in marriage: A growth curve approach. Journal of Marriage and Family.

[CR38] Lovibond SH, Lovibond PF (1995). Manual for the Depression Anxiety Stress Scales.

[CR39] Maisel NC, Karney BR (2012). Socioeconomic status moderates associations among stressful events, mental health, and relationship satisfaction. Journal of Family Psychology.

[CR40] Markman, H. J., Renick, M. J., Floyd, F. J., Stanley, S. M., & Clements, M. (1993). Preventing marital distress through communication and conflict management training: A 4-and 5-year follow-up. *Journal of Consulting and Clinical Psychology*, *61*, 70–77. 10.1037//0022-006x.61.1.70 8450110

[CR41] McRae CS, Overall NC, Henderson AME, Low RST, Chang VT (2021). Parents’ distress and poor parenting during a COVID-19 lockdown: The buffering effects of partner support and cooperative coparenting. Developmental Psychology.

[CR42] Mikulincer, M., & Shaver, P. R. (2016). *Attachment in adulthood: Structure, dynamics, and change* (2nd ed.). Guilford.

[CR43] Muthén, L. K., & Muthén, B. O. (1998–2017). *Mplus user’s guide* (8th ed.). Muthén & Muthén.

[CR44] Neff LA, Karney BR (2017). Acknowledging the elephant in the room: How stressful environmental contexts shape relationship dynamics. Current Opinion in Psychology.

[CR45] Ogolsky, B. G., & Monk, J. K. (Eds.). (2019). *Relationship maintenance: Theory, process, and context*. Cambridge University Press.

[CR46] Overall NC, McNulty JK (2017). What type of communication during conflict is beneficial for intimate relationships?. Current Opinion in Psychology.

[CR47] Overall NC, Chang VT, Pietromonaco PR, Low RS, Henderson AM (2022). Partners’ attachment insecurity and stress predict poorer relationship functioning during COVID-19 quarantines. Social Psychological and Personality Science.

[CR48] Pierce, M., McManus, S., Hope, H., Hotopf, M., Ford, T., Hatch, S. L., & Abel, K. M. (2021). Mental health responses to the COVID-19 pandemic: a latent class trajectory analysis using longitudinal UK data. *The Lancet Psychiatry, 8*, 610–619. 10.1016/S2215-0366(21)00151-6 PMC9764381 33965057

[CR49] Pietromonaco PR, Overall NC (2021). Applying relationship science to evaluate how the COVID-19 pandemic may impact couples’ relationships. American Psychologist.

[CR50] Pietromonaco PR, Overall NC (2022). Implications of social isolation, separation, and loss during the COVID-19 pandemic for couples' relationships. Current Opinion in Psychology.

[CR51] Randall AK, Bodenmann G (2017). Stress and its associations with relationship satisfaction. Current Opinion in Psychology.

[CR52] Reis, H. T., & Clark, M. S. (2013). Responsiveness. In J. A. Simpson & L. Campbell (Eds.), *Oxford library of psychology. The Oxford handbook of close relationships* (p. 400–423). Oxford University Press.

[CR53] Righetti, F., Faure, R., Zoppolat, G., Meltzer, A., & McNulty, J. (2022). Factors that contribute to the maintenance or decline of relationship satisfaction. *Nature Reviews Psychology*, *1*(3), 161–173.

[CR54] Rost J, Petermann F, Eid M (2006). Latent-Class-Analyse [Latent Class Analysis]. Handbuch der Psychologischen Diagnostik.

[CR55] Russell BS, Tambling RR, Horton AL, Hutchison M, Tomkunas AJ (2021). Clinically significant depression among parents during the COVID-19 pandemic: Examining the protective role of family relationships. Couple and Family Psychology: Research and Practice.

[CR56] Russell D, Peplau LA, Cutrona CE (1980). The revised UCLA Loneliness Scale: Concurrent and discriminant validity evidence. Journal of Personality and Social Psychology.

[CR57] Sels, L., Galdiolo, S., Gaugue, J., Geonet, M., Verhelst, P., Chiarolanza, C., Randall, A. K., Verhofstadt, L. (2022 Jan 10). Intimate relationships in times of COVID-19: a descriptive study of Belgian partners and their perceived well-being. *Psychologica Belgica*, *62*(1):1–16. 10.5334/pb.1088. PMID: 35087676; PMCID: PMC8757384. 10.5334/pb.1088 PMC8757384 35087676

[CR58] Sillars A, Vangelisti AL, Vangelisti AL, Perlman D (2006). Communication: Basic properties and their relevance to personal relationships. The Cambridge handbook of personal relationships.

[CR59] Waddell, N., Overall, N. C., Chang, V. T., & Hammond, M. D. (2021). Gendered division of labor during a nationwide COVID-19 lockdown: Implications for relationship problems and satisfaction. *Journal of Social and Personal Relationships*, *38*(6), 1759–1781.

[CR60] Westrupp, E. M., Greenwood, C. J., Fuller-Tyszkiewicz, M., Olsson, C. A., Sciberras, E., Mikocka-Walus, A., & Youssef, G. J. (2021). Parent and child mental health trajectories April 2020 to May 2021: strict lockdown versus no lockdown in Australia. *Australian & New Zealand Journal of Psychiatry*, 00048674211065365. 10.1177/00048674211065365 34930045

[CR61] Westrupp, E. M., Karantzas, G., Macdonald, J. A., Olive, L., Youssef, G., Greenwood, C. J., & Olsson, C. A. (2020). Study Protocol for the COVID-19 Pandemic Adjustment Survey (CPAS): A Longitudinal Study of Australian Parents of a Child 0–18 Years. *Frontiers in Psychiatry, 11*. 10.3389/fpsyt.2020.555750 PMC7488979 33110413

[CR62] Westrupp EM, Macdonald J, Evans S (2022). Developmental gains and losses during parenthood. Current Opinion in Psychology.

[CR63] Westrupp EM, Marshall EM, Bennett C, Benstead M, King G, Karantzas GC, Mogilski J, Shackeford T (2022). Parenting and relationship maintenance. The Oxford Handbook of Evolutionary Psychology and Romantic Relationships.

[CR64] Williamson, H. C. (2020). Early effects of the COVID-19 pandemic on relationship satisfaction and attributions. *Psychological Science*, *31*(12), 1479–1487. 10.1177/0956797620972688 PMC7797601 33151125

[CR65] Yum, Y. O. (2003). The relationships among loneliness, self/partner constructive maintenance behavior, and relational satisfaction in two cultures. *Communication Studies*, *54*(4), 451–467.

