# Supplementary material for: Predicting the Trajectories of Parents’ Relationship Well-Being During COVID-19 Lockdowns and Beyond: a Vulnerability, Stress and Adaptation Model Perspective
Source: Prev Sci. 2023 Mar 28;25(2):213–29. doi: 10.1007/s11121-023-01498-1 (PMC10043525; doi:10.1007/s11121-023-01498-1)
Supplement: Supplementary file 1 — Supplementary file1 (DOCX 235 KB) [file 11121_2023_1498_MOESM1_ESM.docx]

**Supplementary Materials**

| 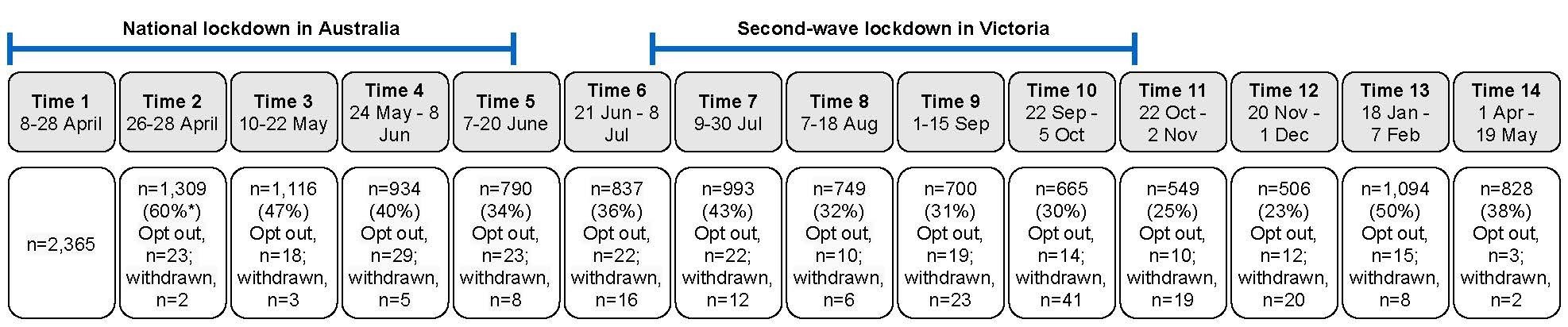 |
| --- |
| Online Resource 1. Schema showing participant flow in the COVID-19 Pandemic Adjustment Survey (CPAS). Note: The eligible sample for Time 2 was N=2,200 given that 165 participants recruited late in Time 1 were not invited to participate at Time 2 |

Online Resource 2.

Comparisons of Wald Test of Parameter Constraints within Victorian and Non-Victorian profiles groups.

|  |  |  | Wald Test Value | df | *p* |
| --- | --- | --- | --- | --- | --- |
| **Relationship Satisfaction** | | | | | |
| *Within Vic group* | | | | | |
|  | Profile 1 | Profile 2 | 295.50 | 4 | < .0001 |
|  |  | Profile 3 | 43.30 | 4 | < .0001 |
|  | Profile 2 | Profile 3 | 69.49 | 4 | < .0001 |
| *Within Non-Vic group* | | | | | |
|  | Profile 1 | Profile 2 | 357.85 | 4 | < .0001 |
|  |  | Profile 3 | 40.82 | 4 | < .0001 |
|  | Profile 2 | Profile 3 | 129.80 | 4 | < .0001 |
| *Between Vic and Non-Vic* | | | | | |
|  | Profile 1 | Profile 1 | 15.54 | 4 | .004 |
|  | Profile 2 | Profile 2 | 3.06 | 4 | .55 |
|  | Profile 3 | Profile 3 | 4.67 | 4 | .32 |
| **Loneliness** | | | | | |
| *Within Vic group* | | | | | |
|  | Profile 1 | Profile 2 | 239.48 | 4 | < .0001 |
|  |  | Profile 3 | 264.39 | 4 | < .0001 |
|  | Profile 2 | Profile 3 | 2.61 | 4 | .63 |
| *Within Non-Vic group* | | | | | |
|  | Profile 1 | Profile 2 | 273.25 | 4 | < .0001 |
|  |  | Profile 3 | 206.83 | 4 | < .0001 |
|  | Profile 2 | Profile 3 | 2.54 | 4 | .64 |
| *Between Vic and Non-Vic groups* | | | | | |
|  | Profile 1 | Profile 1 | 17.13 | 4 | .002 |
|  | Profile 2 | Profile 2 | 5.00 | 4 | .29 |
|  | Profile 3 | Profile 3 | 8.58 |  | .07 |

Table S2.

Significance test for comparisons of growth parameters within Victorian and Non-Victorian profile groups (*p*-value for Wald Test of Parameter Constraints).

|  |  |  | Intercept | Linear | Quadratic | Cubic |
| --- | --- | --- | --- | --- | --- | --- |
| **Relationship Satisfaction** | | | | | | |
| *Within Vic group* | | | | | | |
|  | Profile 1 | Profile 2 | < .0001 | .18 | .12 | .34 |
|  |  | Profile 3 | < .0001 | .26 | .14 | .35 |
|  | Profile 2 | Profile 3 | < .0001 | .85 | .68 | .75 |
| *Within Non-Vic group* | | | | | | |
|  | Profile 1 | Profile 2 | < .0001 | .27 | .71 | .79 |
|  |  | Profile 3 | < .0001 | .77 | .004 | .002 |
|  | Profile 2 | Profile 3 | < .0001 | .54 | .001 | .002 |
| *Between Vic and Non-Vic groups* | | | | | | |
|  | Profile 1 | Profile 1 | .002 | .16 | .004 | .03 |
| **Loneliness** | | | | | | |
| *Within Vic group* | | | | | | |
|  | Profile 1 | Profile 2 | < .0001 | .45 | .51 | .47 |
|  | Profile 1 | Profile 3 | < .0001 | .44 | .83 | .88 |
| *Within Non-Vic group* | | | | | | |
|  | Profile 1 | Profile 2 | < .0001 | .79 | .43 | .61 |
|  | Profile 1 | Profile 3 | < .0001 | .48 | .57 | .47 |
| *Between Vic and Non-Vic groups* | | | | | | |
|  | Profile 1 | Profile 1 | .002 | .002 | .11 | .69 |
